# Extreme exploitation in Southeast Asia waters: Challenges in progressing towards universal health coverage for migrant workers

**DOI:** 10.1371/journal.pmed.1002441

**Published:** 2017-11-22

**Authors:** Rapeepong Suphanchaimat, Nareerut Pudpong, Viroj Tangcharoensathien

**Affiliations:** 1 International Health Policy Program, Ministry of Public Health, Nonthaburi, Thailand; 2 Banphai Hospital, Khon Kaen, Thailand; 3 Healthcare Accreditation Institute (Public Organization), Ministry of Public Health, Nonthaburi, Thailand

## Abstract

Rapeepong Suphanchaimat and colleagues present the plight of migrant workers in the fishing industry in Southeast Asia and discuss challenges in providing for their health and safety.

**Summary points**

- Labour exploitation and enslavement of sea workers have caught significant political attention in many Southeast Asian countries in recent years. These human rights violations are complicated by human trafficking syndicates, economic disparities between countries in the region, weak rule of law, inadequate labour inspection and protections, poor access to healthcare, and corruption.
- Although some Southeast Asian nations attempt to protect the health and well-being of “everyone” on their soil by introducing health insurance policies, there remain unsolved implementation challenges.
- Effectively combating extreme labour exploitation requires a collective effort from all concerned stakeholders, seamless collaboration across countries, and long-term comprehensive mechanisms to prevent further abusive treatments; this is particularly relevant with a highly mobile population like migrant seafarers.

## Introduction

Southeast Asia is one of the regions with the highest rates of population mobility in the world, largely due to worker migration [1]. After the 10 member states of the Association of Southeast Asian Nations (ASEAN), namely, Brunei, Cambodia, Indonesia, Lao PDR, Malaysia, Myanmar, Philippines, Singapore, Thailand, and Vietnam, committed to full economic and social integration with the implementation of the ASEAN Economic Community (AEC) in 2015, labour migration increased markedly [2]. However, there is significant diversity across the 10 member states in their economic and political structures and their health systems [3]. In 2015, the gross national income (GNI) per capita in Singapore was US$52,090, almost 50 times that of Cambodia at US$1,070 [4]. The minimum daily wage in Thailand (US$10) is more than 3 times that in Myanmar (US$2.80) [5]. Such differences have contributed to massive flows of workers from less to more affluent countries, such as Malaysia, Singapore, and Thailand, which have shortages of low-skilled workers needed by their fast-growing economies. In particular, workers for the so-called 3D jobs (dirty, dangerous, and demeaning) in industries such as fishery and construction are in very high demand [6,7].

This article examines the conditions that foster and perpetuate the exploitation of migrant workers in Southeast Asia and addresses the challenge of meeting the health needs of exploited workers. In particular, the exploitation of those working in the fishing industry is so extreme that it has been characterized by some as “sea slavery”. A commercial fishing industry has existed in Southeast Asia since 1850. In the 1900s, it experienced rapid growth to serve a growing population and to fuel regional export markets [8], and it continues in this century to demand a low-paid, regular workforce. Commercial fishing is amongst the most dangerous occupations practiced in the region, involving long work shifts, physically demanding tasks, poor availability and use of protective equipment, inexperienced crew, and high injury rates. Adverse weather and night shifts also pose a high risk of accidents [9].

In extreme labour exploitation, a person cannot refuse a work assignment or leave because of threats, violence, coercion, deception, or abuse of power [10]. Such a situation falls in the concept of “modern slavery”. The International Labour Organization (ILO) indicates that in 2016 there were around 40 million victims of modern slavery worldwide. The most prevalent region is Africa (about 7.6 victims for every 1,000 people) followed by Asia-Pacific (about 6.1 victims for every 1,000 people) [11]. In Southeast Asia, around 2.5 million people were caught up in the grip of modern slavery. According to the Global Slavery Index, 4 ASEAN countries (Cambodia, Myanmar, Brunei, and Thailand) are amongst the top 10 Asian nations with the greatest number of people working under conditions of extreme exploitation (Table 1) [10].

**Table 1:** Asian countries with the highest percentage of people working in extreme exploitation.

Country	Estimated percentage of population	Estimated number in the population	Total population
North Korea	4.4	1,100,000	25,155,000
Cambodia	1.6	256,800	15,578,000
India	1.4	18,354,700	1,311,051,000
Pakistan	1.1	2,134,900	188,925,000
Afghanistan	1.1	367,600	32,527,000
Myanmar	1.1	515,100	53,897,000
Bangladesh	1.0	1,531,300	160,996,000
Nepal	0.8	234,600	28,514,000
Brunei	0.8	3,400	423,000
Thailand	0.6	425,500	67,959,000

Source: Minderoo Foundation (2017) [10].

In Southeast Asia, the belief that “healthy migrants” contribute to a “healthy economy” for the whole region has brought political scrutiny to the health of migrant workers. The global movement towards universal health coverage (UHC), gaining momentum from the United Nations Sustainable Development Goals (SDGs) of 2015, has also brought attention to the issue. UHC has the potential to reach all workers, including those in conditions of extreme exploitation, to improve their health and contribute to the protection of their human rights. Extreme labour exploitation is a human rights violation, and the World Health Organization (WHO) has identified the following 3 dimensions in which health and human rights intersect: violations of human rights can lead to serious health outcomes, health policies and programs can promote human rights in their design and implementation, and vulnerability to ill health can be minimized by taking steps to fulfill a country’s obligations to human rights [12].

### Factors facilitating and perpetuating the extreme exploitation of sea workers

Exploitation of workers in Southeast Asian waters is not new, but it was the Rohingya maritime crisis in 2015 that brought the problem to the world’s attention. Not all Rohingyas voluntarily fled the conflicts in their country of origin [13]—many were smuggled or trafficked, that is, recruited and transported forcibly or under threat of force. In 2012, the ILO estimated that there were about 11.7 million trafficked persons in Southeast Asia, a status that leaves them vulnerable to extreme exploitation [14]. Even those people who migrate of their own volition may arrive without valid travel documents, leaving them at risk of exploitation.

Work-related hazards and violence are common experiences faced by undocumented migrants, particularly sea workers [15]. Employment abuses exacerbate the difficulties of sea workers and include confiscation of workers’ identification documents (such as passport or work permit) by employers, delayed payments, a lack of labour protection, and the absence of clear channels to report abusive acts to government officials [16]. In many countries, the ability of labour inspectors to identify and support sea workers in need of assistance still falls short of international standards [17]. On-site labour inspection is challenging, as it is difficult to witness the maltreatment of employers on boat crews offshore. Most sea workers spend long periods on ships, making it hard to report poor working conditions without facing intimidation and coercion from employers [17,18]. A 2013 survey of 596 fishermen (including Thai workers and Cambodian and Burmese migrants) in Thailand by the ILO (2013) found that around 14% of the fishermen did not complain about serious human rights violations to labour inspectors for fear of causing problems with their employers [19]. About 7% reasoned that they neither knew to whom or where they could complain nor trusted that such a complaint would help improve their work conditions [19]. There are also reports of officials who might be complacent in response to, or complicit with, labour abuses and trafficking [20].

Brokers play a central role in migrant labour markets. Some assist migrants and employers in a lawful manner. However, as revealed by Greenpeace in 2016, some brokers work as a syndicate and become involved in almost all stages of the employment process, from falsifying attractive work conditions and high wages to processing fake entry documents [17,18,21]. Given the scale of unscrupulous broker activity and illegal trafficking syndicates, which may be aided by corrupt government officials, the chance of a prospective migrant worker meeting a “good broker” is unpredictable [22].

Despite the fact that some countries conduct health screening measures before issuing work permits, sea workers still experience poor health. A survey of 406 fishermen in Malaysia reported that around 12% of the participants were HIV positive and more than one third had a history of using injection drugs [23]. These problems were even more apparent amongst trafficked sea workers due to their precarious legal status and excessive work hours offshore, lasting weeks or even months at a time without adequate respite [19,24].

The “Declaration on the Protection and Promotion of the Rights of Migrant Workers”, launched at the 2007 ASEAN Summit, stipulated the obligations of sending and receiving countries to protect the rights and dignity of migrant workers and their families [25]. These ideas became part of the strategic objectives enshrined in the ASEAN Socio-Cultural Community (ASCC) Blueprint [26]. Yet, it is doubtful whether or to what extent these written statements have been implemented. Furthermore, discussions leading up to the Declaration were largely around skilled migrants, not low-skilled migrant workers and their dependants, let alone trafficked and exploited people [1]. In addition, various legal interpretations exist regarding which groups of nonnationals should be covered, including undocumented migrants, dependants of undocumented migrants, victims of human trafficking, and people who have been denied formal nationality by the state they live in or the state they have fled (such as Rohingya stateless people) [27]. Moreover, the 2007 ASEAN Declaration lacks legal instruments to sanction countries in which the rights of migrants are breached.

### UHC in Southeast Asia—The unsolved challenges for exploited sea workers

For most ASEAN governments, apart from the desire for a healthy workforce to support the economy, migrant worker health is of concern to ensure health security for their citizens by screening for communicable diseases, such as tuberculosis and filariasis, commonly transmitted by migrants. Other aspects of labour protection, such as occupational injury treatment, are often overlooked [1].

One concrete measure that has been taken to protect the health of migrant workers is a public insurance system. Thailand has introduced a health card scheme for undocumented migrants, but its implementation has faced challenges. The scheme is de facto only available to undocumented workers and their dependants from Cambodia, Lao PDR, and Myanmar under the condition that they must have registered with the Thai government before the end of 2014 under the One Stop Service (OSS) measure [27]. This regulation has created obstacles for newly identified undocumented trafficked migrants wishing to register. Furthermore, the scheme did not clearly specify who (between migrants and employers) is responsible for paying for the annual fees of the health card [27]. In practice, some employers paid the fee for health cards and other essential documents in advance but then deducted this cost from their employees' payroll [20,28]. Some employers confiscated their workers' travel documents or work permits to prevent workers from changing jobs and to ensure that they worked with them long enough to recoup their costs [20]. Health insurance is a particularly complex matter for migrant seafarers. Some found the premiums too expensive and refused to buy the insurance card. The long time spent offshore limited their ability to use the insurance. Some were not even aware of the existence of the scheme [27].

Malaysia provides another troubling example of unmet challenges. Foreign workers were required to take up the compulsory insurance scheme, the Skim Perlindungan Insurans Kesihatan Pekerja Asing (SPIKPA). The scheme is financed through a premium paid by employees and is operated by 28 private insurance carriers. Insurees are required to show their private insurance cards to hospital staff to receive exemption from up-front cash payments for services. However, undocumented migrants who fail to register with the government are not allowed to buy the insurance. Besides, many employers kept migrants' health cards and passports, making it difficult for them to avail themselves of health services [1]. The poor design of a health scheme and unethical practices of employers are important factors that hinder healthcare access amongst migrants, especially undocumented and trafficked ones.

## Conclusions and recommendations

Implementing UHC for migrant and exploited sea workers is a worthy goal, but it is insufficient to protect the health of sea workers who are in or vulnerable to conditions of extreme exploitation. Structural problems that make exploitation possible are the highest priority. Addressing these problems requires the collective effort of multiple stakeholders. It is impossible for a single country to tackle extreme labour exploitation in the fishing industry alone, because migrant seafarers travel across national boundaries. All ASEAN nations need to take dedicated and bold actions to combat corruption that ignores or fuels exploitation and understand that labour exploitation can befall any group of people in a position of vulnerability. This concept of vulnerability to exploitation will broaden the scope of actions of all governments, moving from the sporadic arrest of unscrupulous officials to tackling all structural problems that provide opportunities for corruption or create conditions that make people vulnerable to exploitation. For instance, the registration process for undocumented migrants should take place without financial barriers, thereby minimizing room for dishonest officials, employers, and brokers to take these benefits from migrants. Governments should create easily accessible means for migrants, especially undocumented or trafficked workers, to voice their concerns if they experience maltreatment from employers and officials. Because trafficked workers and those in vulnerable situations typically fear reporting corrupt behaviour of officials via routine government channels, governments can benefit from working with the media and nongovernmental organizations (NGOs) in establishing vigilance and reporting channels. Governments should regard civic groups and NGOs as allies, releasing information on trafficking so they can fight together to end these practices, rather than viewing such groups and organizations as enemies who will damage the nation’s reputation. In addition, ad hoc humanitarian rescues, such as the provision of food and shelter to abused victims, should not be seen as the only solution; long-term and comprehensive intersectoral measures are urgently required.

On the pathway towards achieving UHC for everybody, several barriers to migrants’ accessing health services need to be overcome. Governments must ensure adequate and sustainable resources to provide basic health benefits to undocumented migrants and their dependants to prevent financial catastrophe. Nationality verification processes for undocumented and trafficked persons need to be streamlined and accelerated. Mechanisms should be put in place to ensure the compatibility and transferability of insurance coverage from home country insurance systems. ASEAN governments need to ensure that seafood entrepreneurs, such as owners of restaurants and seafood processing factories, in their country are not involved in forced labour or exploitation. Migrant workers, despite their vulnerable status, should be positioned as active agents in policy design, with greater negotiating power to ensure better working conditions. The contributions that migrant workers make to a country’s economy, especially in the context of labour shortages, should be made more evident to the public and to policy makers so that public discourse can shift policy towards better protection of migrants. Measures such as these have the potential to collectively bring about the gradual realisation of UHC throughout Southeast Asia, not only for exploited or trafficked sea workers but for all migrant workers. Although there is a long way to go, the global momentum towards UHC driven by the SDGs, along with the pledge of all governments in ASEAN to protect the health of everybody in the region, will make progress possible if words are put into actions by all concerned.

## Abbreviations

AEC: ASEAN Economic Community
ASCC: ASEAN Socio-Cultural Community
ASEAN: Association of Southeast Asian Nations
GNI: gross national income
ILO: International Labour Organization
NGO: nongovernmental organization
OSS: One Stop Service
SDG: Sustainable Development Goal
SPIKPA: Skim Perlindungan Insurans Kesihatan Pekerja Asing
UHC: universal health coverage
WHO: World Health Organization
